# On the Ultrastructure and Function of Rhogocytes from the Pond Snail *Lymnaea stagnalis*

**DOI:** 10.1371/journal.pone.0141195

**Published:** 2015-10-21

**Authors:** Maria Kokkinopoulou, Lisa Spiecker, Claudia Messerschmidt, Mike Barbeck, Shahram Ghanaati, Katharina Landfester, Jürgen Markl

**Affiliations:** 1 Institute of Zoology, Johannes Gutenberg University, 55099 Mainz, Germany; 2 Max Planck Institute for Polymer Research, 55128 Mainz, Germany; 3 Institute of Pathology, University Medical Center of the Johannes Gutenberg University Mainz, 55101 Mainz, Germany; George Washington University School of Medicine and Health Sciences, UNITED STATES

## Abstract

Rhogocytes, also termed “pore cells”, occur as solitary or clustered cells in the connective tissue of gastropod molluscs. Rhogocytes possess an enveloping lamina of extracellular matrix and enigmatic extracellular lacunae bridged by cytoplasmic bars that form 20 nm diaphragmatic slits likely to act as a molecular sieve. Recent papers highlight the embryogenesis and ultrastructure of these cells, and their role in heavy metal detoxification. Rhogocytes are the site of hemocyanin or hemoglobin biosynthesis in gastropods. Based on electron microscopy, we recently proposed a possible pathway of hemoglobin exocytosis through the slit apparatus, and provided molecular evidence of a common phylogenetic origin of molluscan rhogocytes, insect nephrocytes and vertebrate podocytes. However, the previously proposed secretion mode of the respiratory proteins into the hemolymph is still rather hypothetical, and the possible role of rhogocytes in detoxification requires additional data. Although our previous study on rhogocytes of the red-blooded (hemoglobin-containing) freshwater snail *Biomphalaria glabrata* provided much new information, a disadvantage was that the hemoglobin molecules were not unequivocally defined in the electron microscope. This made it difficult to trace the exocytosis pathway of this protein. Therefore, we have now performed a similar study on the rhogocytes of the blue-blooded (hemocyanin-containing) freshwater snail *Lymnaea stagnalis*. The intracellular hemocyanin could be identified in the electron microscope, either as individual molecules or as pseudo-crystalline arrays. Based on 3D-electron microscopy, and supplemented by *in situ* hybridization, immunocytochemistry and stress response experiments, we provide here additional details on the structure and hemocyanin biosynthesis of rhogocytes, and on their response in animals under cadmium and starvation stress. Moreover, we present an advanced model on the release of synthesized hemocyanin molecules through the slit apparatus into the hemolymph, and the uptake of much smaller particles such as cadmium ions from the hemolymph through the slit apparatus into the cytoplasm.

## Introduction

**Fig 1:**
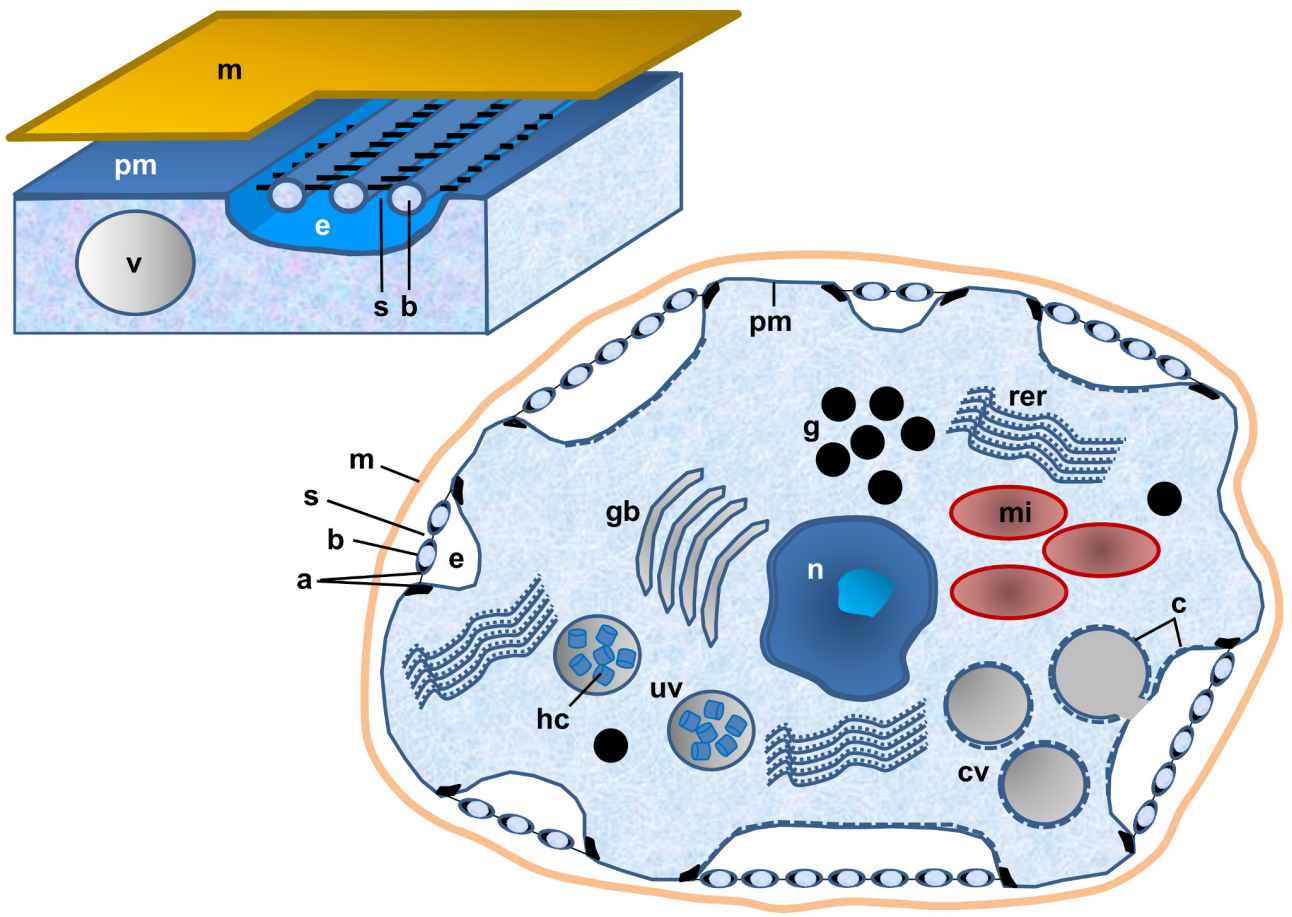
Characteristic features of gastropod rhogocytes. Note the plasma membrane (pm), nucleus (n), mitochondria (mi), Golgi bodies (gb), rough endoplasmic reticulum (rer), electron-dense granula (g), vesicles (v), uncoated vesicles (uv), coated vesicles (cv), coat (c), hemocyanin molecules (hc), extracellular lacunae (e), cytoplasmic bars (b), diaphragmatic slits (s), enveloping lamina of extracellular matrix (m), electron-dense material (a) at the edges of the bars. The slit apparatus resembles a gully grate (left scheme), but most often transversal cuts through the cytoplasmic bars are seen (right scheme).

Rhogocytes are characteristic cells that occur either free in the hemolymph or embedded in the connective tissue of gastropods and other members of the phylum Mollusca. They are also known as pore cells, Leydig cells, cellule nucale, blasenzellen or brown cells (for review, see [1]). They vary greatly in size (2–30 μm) and shape (elongated, round, irregular), but are identified in tissue sections even at low magnification due to their typical structure, notably a well-developed endoplasmic reticulum and a large nucleus [2,3]. There is no observable cell polarity. Rhogocytes show a single nucleolus, many electron-dense granula and much lighter secretory vesicles, as well as mitochondria and Golgi bodies (Fig 1). Rhogocytes are often found in clusters, but a direct cell-cell contact is prevented by a lamina of extracellular matrix that envelopes each rhogocyte.

As their most typical structural feature, rhogocytes are characterized by invaginations of the plasma membrane forming extracellular lacunae (see Fig 1). In electron micrographs these lacunae are either empty or contain granular material and sometimes even vesicles [3]. The lacunae are bridged by cytoplasmic bars, forming diaphragmatic slits (the Greek word *rhogos* means “slit”). This “slit apparatus” resembles a gully grate (see Fig 1) and should function as a molecular sieve of *ca*. 20 nm width (see [1–4], and literature cited therein). Ultrastructurally, it corresponds to the molecular sieve found in insect nephrocytes and mammalian podocytes [1,5,6]. Nephrocytes and podocytes share similar proteins, notably nephrin and actin, in association with the slit apparatus [7]. In a previous paper we collected evidence that both proteins are also present in the slit apparatus of *Biomphalaria glabrata* rhogocytes [3]. This molecular result supported the long-standing hypothesis of a common phylogenetic origin of the three cell types [1].

Rhogocytes synthesize the hemolymph respiratory proteins hemocyanin and hemoglobin [3,4,8]. Moreover, they are involved in transport and storage of nutrients [1,2, 9], participate in calcium mobilization for shell formation [10], partake in defense systems [11] and act in heavy metal detoxification [12,13]. With respect to the putative function of the slit apparatus as a molecular sieve, neither the nature of the filtered particles nor their migration direction is well understood [1]. Recently, we provided evidence from *B*. *glabrata* that the hemoglobin molecules synthesized by rhogocytes are exported through the slit apparatus into the hemolymph by merocrine secretion [3]. Moreover, we proposed that heavy metal ions freely diffuse from the hemolymph through the slits into the extracellular lacunae, are then endocytosed by coated vesicles and stored in the electron-dense granula [3]. However, although this red iron-containing hemoglobin is a large multimer measuring 25 nm across [14], its identification in electron micrographs of tissue sections remains uncertain, because it lacks a characteristic profile [3].

In gastropods, hemoglobin occurs specifically in members of the family Planorbidae, whereas most other gastropods possess hemocyanin. Hemocyanin is blue, contains copper in its active site and serves as extracellular respiratory protein in the hemolymph. Its accumulation in large vesicles of rhogocytes has been noted in the electron microscope [15–18], but it remained open as to whether it was synthesized or degraded by these cells. Its biosynthesis in rhogocytes was ultimately confirmed by *in situ* hybridization in the vetigastropods *Haliotis tuberculata* and *Megathura crenulata* [4,8]. Gastropod hemocyanin is a cylindrical didecamer of 400 kDa subunits and measures *ca*. 35 nm across; it is structurally defined in detail [19–21].

In our present study, we took advantage of the fact that due to their circular top view and rectangular side view, hemocyanin didecamers are often clearly identified in electron micrographs of tissue sections. The pond snail *Lymnaea stagnalis*, an abundant freshwater snail, has hemocyanin, and its biosynthesis in rhogocytes has been proposed [15,18,22]. In the present study, our aim was to reveal greater detail of the ultrastructure of rhogocytes, to monitor cellular changes caused by starvation stress and heavy metal (notably cadmium) exposure, and to trace the excretion pathway of hemocyanin from the cytoplasm through the slit apparatus into the hemolymph.

## Results

### Rhogocytes as the site of hemocyanin biosynthesis in *L*. *stagnalis*

**Fig 2:**
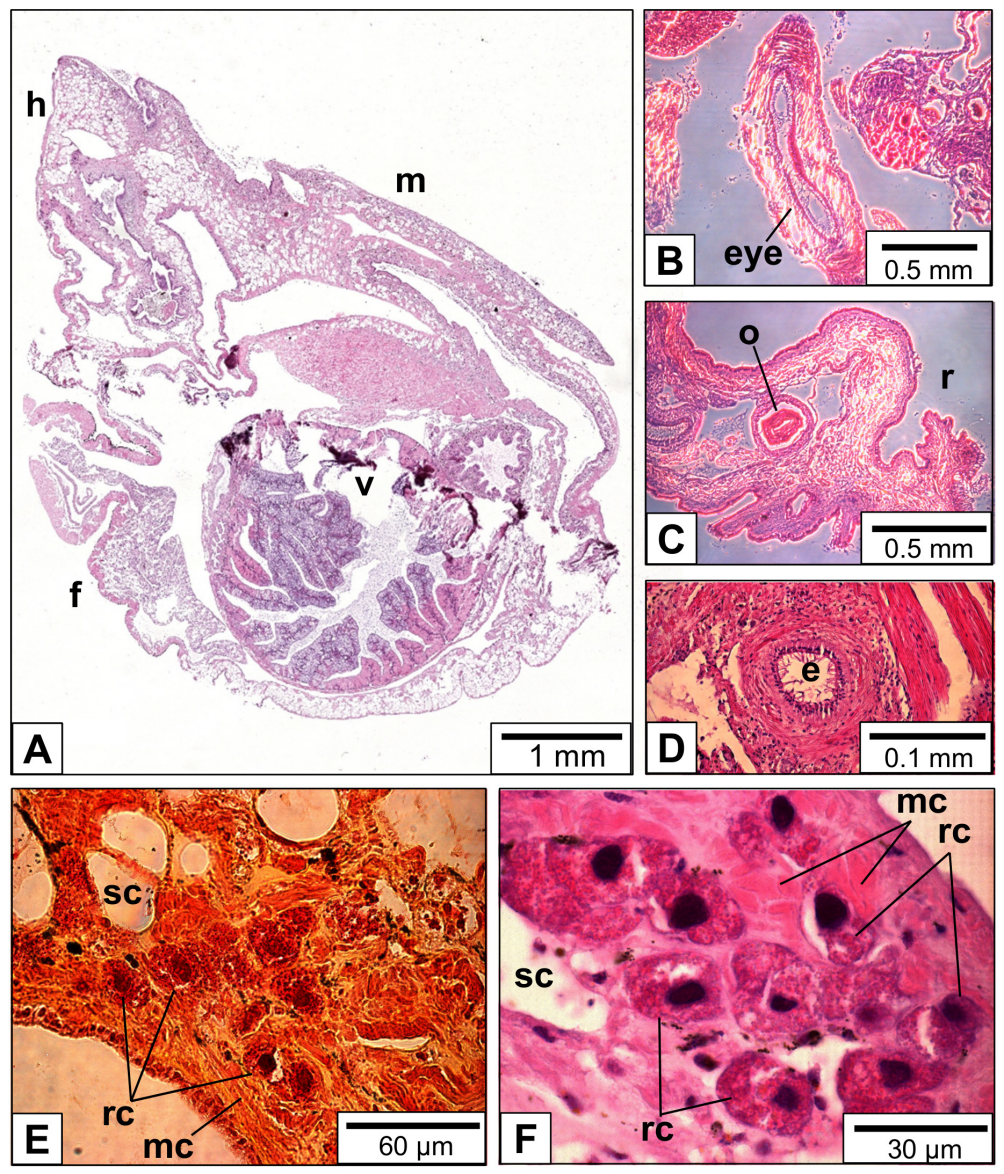
Light microscopy of paraffin-embedded tissue sections of *L*. *stagnalis*. (**A**) Total scan of a diagonal cut through the animal (h, head; m, mantle; f, foot; v, visceral sac). (**B**-**D**) Head tissue sections; prominent structures such as eye, odontophore (o), radula (r) and esophagus (e) could be identified. (**E**, **F**) Mantle tissue sections; note that besides muscle cells (mc) and secretory cells (sc), many rhogocytes (rc) are visible. The rhogocytes are identified by their large nucleus and lamellar substructure. Hematoxylin & eosin stain (B-D, F), and Movat´s pentachrome stain (E) was applied.

An individual of *L*. *stagnalis* was entirely cut into tissue sections of 3–5 μm thickness. The slices were stained and studied by light microscopy (Fig 2A). Besides the major body parts, organs such as eye (Fig 2B), odontophore (Fig 2C) and esophagus (Fig 2D) can be defined. Due to their lamellar substructure, rhogocytes were identified (often in clusters) in the connective tissue of mantle and foot (Fig 2E and 2F). Also, muscle cells and secretory cells were identified in such sections. As expected, muscle cells dominated the foot tissue, whereas the mantle tissue contained more secretory cells. The latter contained a pale, homogeneous material that might be amorphous calcium carbonate used for shell formation (see Fig 2E).

**Fig 3:**
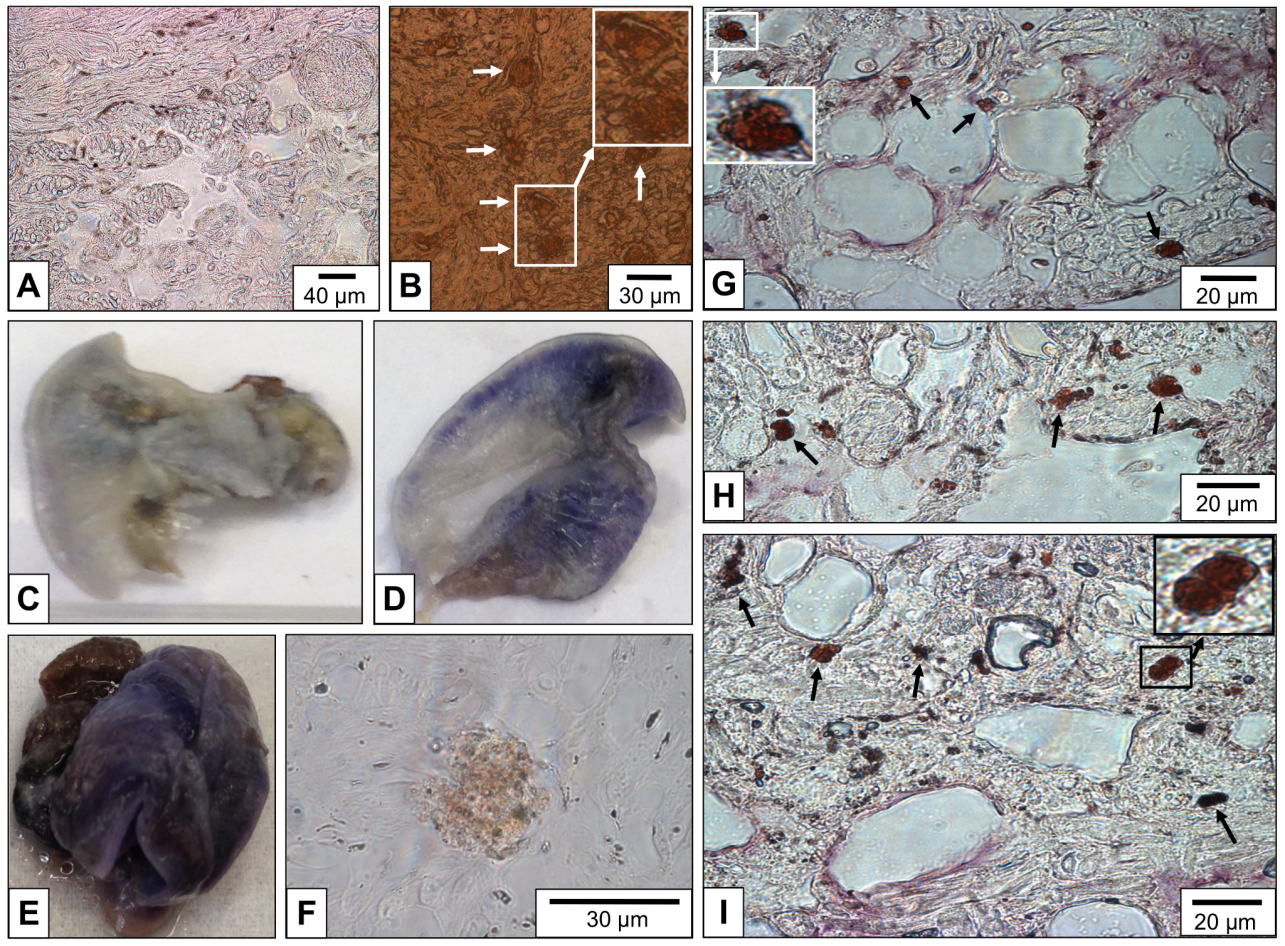
Detection of hemocyanin in *L*. *stagnalis* paraffin-embedded tissues and whole mounts. (**A**, **B**) Immunohistochemistry on paraffin-embedded tissue sections. (**A**) Negative control. (**B)** Rabbit anti-*Aplysia californica* hemocyanin primary antibodies; note strong staining of rhogocytes (arrows). (**C**-**E**) Whole mount *in situ* hybridization of the extracted mantle. (Total length of the animals was *ca*. 2 cm.) (**C**) Negative control, with the DIG-labeled probe omitted. (**D**) 100 ng of the DIG-labeled probe was added; note the blue-purple staining. (**E**) 200 ng of the DIG-labeled probe was added; note deeper blue-purple staining. (**F**) Paraffin-embedded tissue section of the whole mount shown in (E); note specific labeling of a cell morphologically identified as rhogocyte. (**G**-**I**) *In situ* hybridization on paraffin-embedded tissue sections from other *L*. *stagnalis* individuals. The strongly stained cells (arrows) are morphologically identified as rhogocytes.

Immunohistochemistry on *L*. *stagnalis* tissue sections with anti-*Aplysia californica* hemocyanin primary antibodies showed a strong signal in cells morphologically identified as rhogocytes (Fig 3A and 3B, arrows), indicating a comparatively high concentration of this protein in these cells. The hemolymph spaces of the open circulatory system showed a weaker signal, suggesting a lower hemocyanin concentration in the hemolymph compared to the rhogocyte subcellular compartments.

*In situ* hybridization was performed on *L*. *stagnalis* whole mounts, using a DIG-labeled cDNA specific for RNA encoding a fragment of functional unit FU-h of *L*. *stagnalis* hemocyanin. Positive reactions of the mantle tissue were observed (Fig 3C–3E). The labeled mantle tissue shown in Fig 3E was thin sectioned to localize the signal of this reaction more specifically. Indeed, the signal was found in cells that resemble rhogocytes (Fig 3F). The same cDNA probe was used to perform *in situ* hybridization on paraffin-embedded tissue sections. A strong and specific labeling of cells morphologically identified as rhogocytes was obtained (Fig 3G–3I). These experiments confirmed that rhogocytes are the site of hemocyanin biosynthesis in *L*. *stagnalis*.

This is the first direct proof, outside of the Vetigastopoda [4,8], that rhogocytes are indeed the site of hemocyanin biosynthesis in gastropods. It is therefore reasonable to assume that hemocyanin production is a general function of gastropod rhogocytes. Based on electron microscopy, this was proposed some decades ago (for review, see [1]).

### Ultrastructure of *L*. *stagnalis* rhogocytes

**Fig 4:**
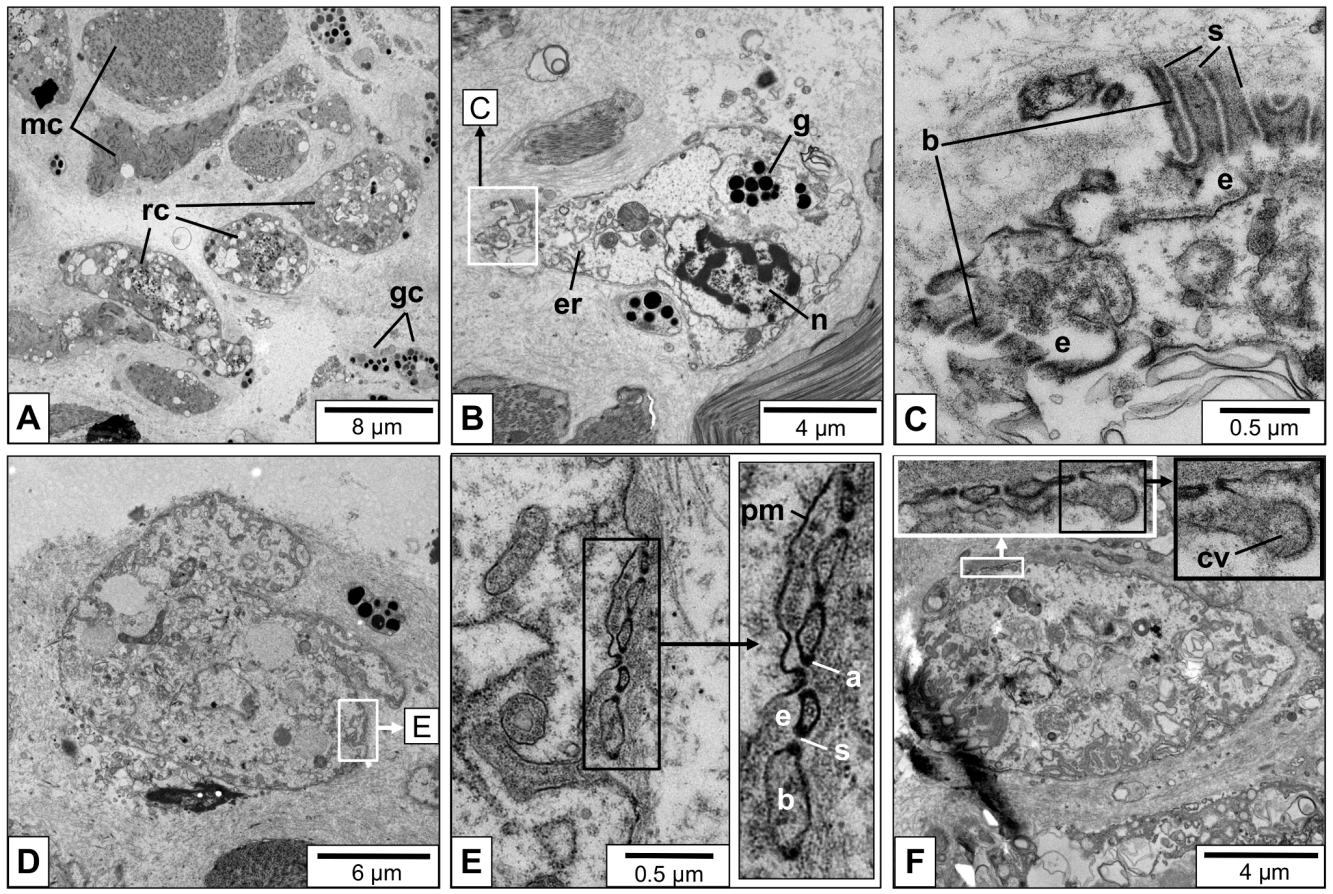
Electron microscopy of *L*. *stagnalis* mantle tissue sections. **(A)** Low magnification image with rhogocytes (rc), muscle cells (mc) and granular cells (gc). **(B)** Medium magnification showing a single rhogocyte; note the big nucleus (n), endoplasmatic reticulum (er) and electron-dense granula (g). The white frame indicates the area magnified in (C). **(C)** Close-up of an area with extracellular lacunae (e), cytoplasmic bars (b), and diaphragmatic slits (s) shown in longitudinal cuts. (**D**) Another single rhogocyte; the frame indicates the area magnified in (E). (**E**) Close-up of the area marked in (D), with a further magnification (frame) presented in the insert. It shows the cytoplasmic bars (b) and diaphragmatic slits (s) in a transversal cut. The extracellular lacunae (e) are filled with granular material. Note at both ends of the cytoplasmatic bars the electron-dense material (a). This material is likely to contain actin (see Fig 7). (**F**) Another rhogocyte. Note the magnified slit apparatus in the (white box), and the coated vesicle (cv) fused with the extracellular lacuna (black box).

In confirmation of early electron microscopical data [2], we found many rhogocytes in the connective tissue of *L*. *stagnalis*, together with muscle cells and granular cells (Fig 4A).The rhogocytes varied in shape and size which, however, might at least partially stem from the plane of section. In Fig 4B, typical features such as a large nucleus, prominent endoplasmic reticulum and numerous electron dense granula are shown. At higher magnification, different views of the extracellular lacunae and the slit apparatus are seen (Fig 4C–4F). In shape, the slit apparatus resembles a gully grate. Consequently, transversal sections are frequent (see Fig 4E), and longitudinal ones are rare (see Fig 4C). The extracellular lacunae are either empty or filled with granular material (see Fig 4E). The plasma membrane adjacent to the extracellular lacunae is often coated, and coated vesicles in open contact with the lacuna are also detected (see Fig 4F).

**Fig 5:**
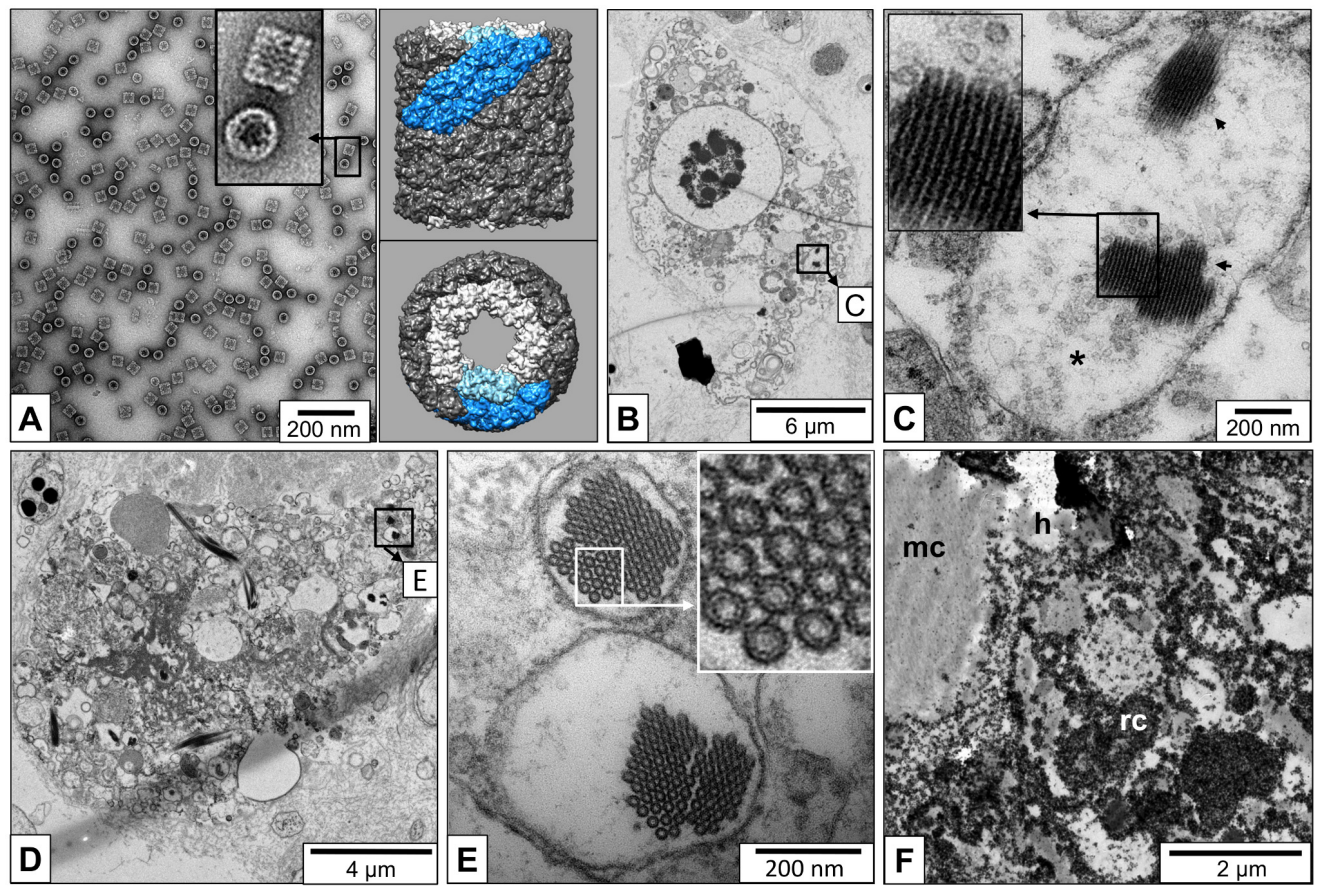
Electron microscopy of hemocyanin and rhogocytes. (**A**) Biochemically isolated, negatively stained *L*. *stagnalis* hemocyanin didecamers. Note the circular top views and the rectangular side views of the protein molecule. The panel shows side and top view of a gastropod hemocyanin didecamer simulated, as 7 Å resolution, using PDB-entry 4BED (deposited by our research group in 2013). Note wall (dark) and collar (light) of the molecule; the ten-fold repeating unit which is a subunit dimer (2x400 kDa) is indicated in blue. **(B)** A rhogocyte, with the area displayed magnified in (C) indicated by a frame. **(C)** Close-up of the framed area in (B), showing an ER compartment with side views of two pseudo-crystalline hemocyanin arrays (arrowheads). Note the surrounding solitary hemocyanin molecules (asterisk). The insert shows an array at higher magnification. (**D**) Another rhogocyte; the frame marks the area magnified in (E). (**E**) Close-up of the framed area in (D), showing two endomembrane compartments with top views of pseudo-crystalline hemocyanin arrays. The insert shows a further enlargement; note details of outer wall and inner collar of the protein cylinder. (**F**) Section of a rhogocyte (rc), a muscle cell (mc) and a hemolymph space (h) in between, labeled with immunogold particles recognizing gastropod hemocyanin. Note that the hemolymph space and compartments of the rhogocyte are strongly marked.

*L*. *stagnalis* hemocyanin biochemically isolated from hemolymph samples and negatively stained has a characteristic appearance in the electron microscope with circular top views and rectangular side views of the cylindrical 35 nm molecules (Fig 5A). Detailed structural knowledge on such gastropod hemocyanin didecamers (composed of twenty subunits of 400 kDa) is available (Fig 5A, right panel). In most *L*. *stagnalis* rhogocytes studied in the electron microscope, compartments of the endomembrane system filled with material resembling hemocyanin were identified (Fig 5B and 5C). The hemocyanin is often present as pseudo-crystalline arrays, surrounded by individual didecamers, both visible as top views and side views (Fig 5C–5E). Similar arrays that represent tubular stacks of didecamers have been previously produced from purified gastropod hemocyanin [23]. In our study, individual hemocyanin stacks and solitary didecamers exhibited a diameter of only 25nm. This smaller size of chemically fixed and embedded didecamers compared to negatively stained didecamers adsorbed on a carbon film (35 nm; see Fig 5A) has also been reported by other authors (e.g. [17,24]).

Immunogold labeling experiments using polyclonal anti-*Aplysia* hemocyanin antibodies indicated the presence of hemocyanin in the hemolymph space between muscle cells and rhogocytes, and also within cytoplasmic compartments of the rhogocytes (Fig 5F).

**Fig 6:**
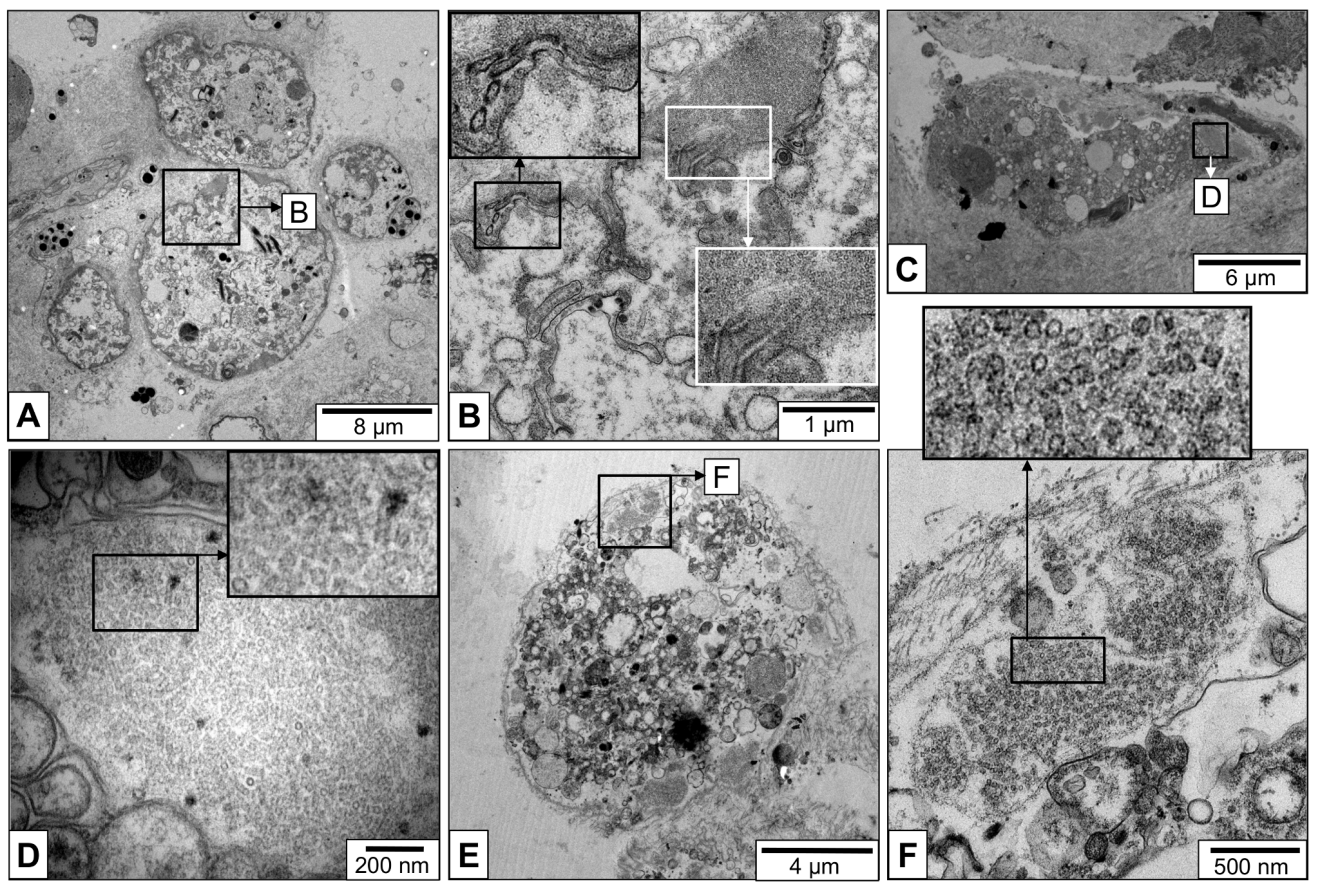
Electron microscopy of mantle rhogocyte regions containing hemocyanin. (**A**) Cluster of rhogocytes in the connective tissue. The framed area is further enlarged in (B). (**B**) Close-up view of the area framed in (A), showing the slit apparatus in transversal cut (black box) and longitudinal cut (white box). Magnifications of both areas are also shown. Note masses of hemocyanin-like particles at both sides of the slit apparatus. (**C**) Rhogocyte showing large peripheral ER cisternae filled with particle. The framed area is further magnified in (D). (**D**) Close-up view of the framed area seen in (C). The insert shows a further enlargement of the region indicated by a black box. Different views of hemocyanin molecules are visible. (**E**) Another rhogocyte containing hemocyanin-rich ER cisternae. The framed area is further magnified in (F). (**F**) Close-up view of the lacuna framed in (E) and filled with hemocyanin. In the magnified box, different views of hemocyanin molecules can be distinguished.

Particles resembling individual hemocyanin molecules were often seen in extracellular lacunae and also outside of the slit apparatus (Fig 6A and 6B). In such cases, it appeared as if the hemocyanin has been released through the slits. Moreover, in the periphery of rhogocytes we frequently observed large ER cisternae containing masses of solitary hemocyanin didecamers (Fig 6C–6F). Such intracellular cisternae were often in close contact with the plasma membrane bordering an extracellular lacuna.

### Evidence for actin and nephrin in the vicinity of the slit apparatus

According to our previous results on *B*. *glabrata* rhogocytes, the proteins actin and nephrin are associated with the slit apparatus [3]. In the molecular sieves of podocytes and nephrocytes, both proteins play an important role in that actin is a component of the cytoplasmic bars and nephrin is a major component of the slit diaphragm [6,7]. Since *B*. *glabrata* rhogocytes produce hemoglobin [3,25] whereas *L*. *stagnalis* rhogocytes produce hemocyanin (see Fig 3), we wanted to determine that they possess the same type of slit apparatus.

**Fig 7:**
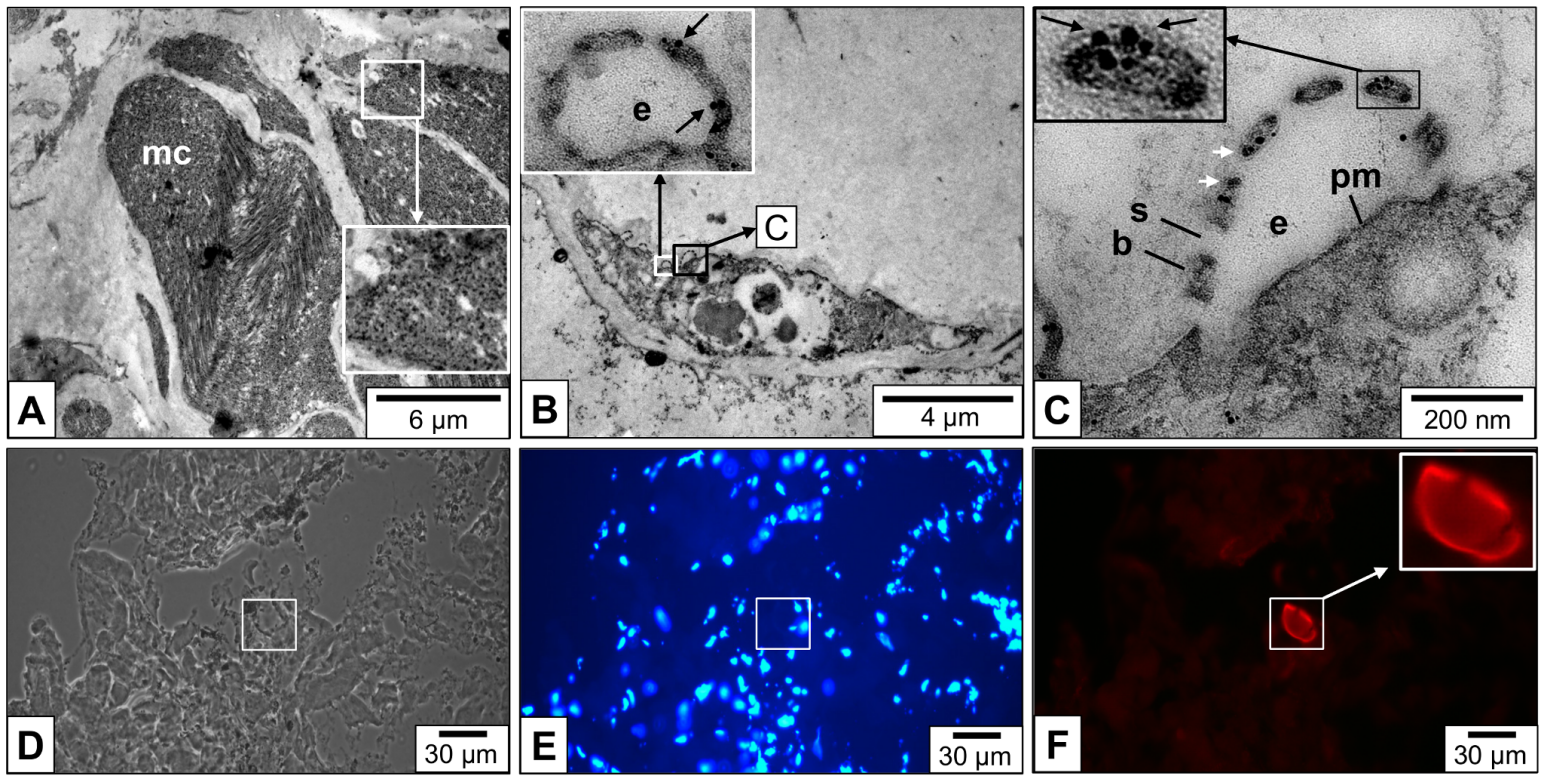
Localization of actin and nephrin in the slit apparatus. (**A**-**C**) Electron micrographs of LR-white embedded mantle tissue sections of *L*. *stagnalis*. (**A**) A group of muscle cells (mc) labeled with a gold-coupled monoclonal anti-actin antibody (black dots in enlarged area). (**B**) A rhogocyte within mantle connective tissue. Note the enlarged area (insert), showing anti-actin immunogold labeling at the edges of the cytoplasmic bars (arrows) covering the extracellular lacuna (e). The black frame indicates the area magnified in (C). (**C**) Close-up of the region framed in (B), showing anti-actin immunogold labeling (arrows) at the cytoplasmic bars (b) close to the diaphragmatic slits (s). Also note boxed enlargement. e, extracellular lacuna; pm, plasma membrane. (**D**-**F**) Immunofluorescence microscopy of a frozen mantle tissue section. (**D**) Phase contrast optics. (**E**) Epifluorescence optics, showing DAPI (diamidino-2-phenylindole) staining to visualize the cell nuclei. (**F**) Epifluorescence optics, showing a positive reaction (red signal) of the periphery of a rhogocyte with polyclonal anti-nephrin antibodies. Also note enlargement.

We used a monoclonal anti-actin primary antibody [26] in immunogold labeling experiments, with *L*. *stagnalis* muscle tissue as a positive control (Fig 7A). Thereby, we collected evidence that actin is major component of the rhogocyte slit apparatus also in *L*. *stagnalis*. Apparently, this protein is localized at the edges of the cytoplasmic bars, at the insertion side of the slits (Fig 7B and 7C), where highly dense material is seen in the electron microscope (see Fig 4E). Polyclonal anti-nephrin antibodies were used on frozen tissue sections of *L*. *stagnalis*. Fluorescence microscopy yielded a strong labeling of the periphery of cells morphologically identified as rhogocytes (Fig 7D–7F). This indicates a role of both, actin and nephrin, in the presumed ultrafiltration process, and suggests that the rhogocytes of red-blooded and blue-blooded gastropods are similar in structure and function.

### Improved optical resolution of the rhogocyte ultrastructure

**Fig 8:**
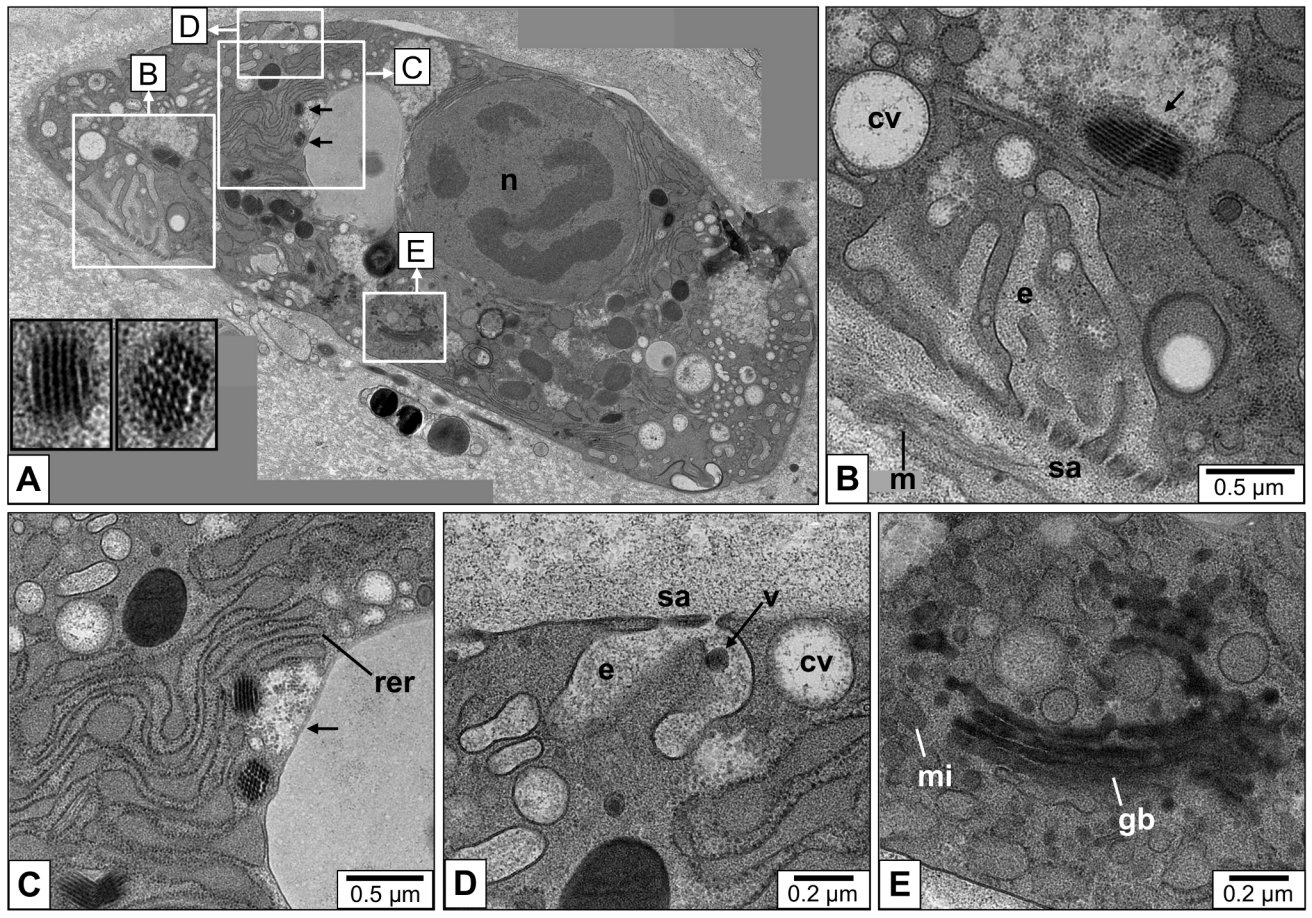
Electron microscopy of mantle tissue sections fixed by high pressure freezing and freeze substitution. (**A**) A single rhogocyte composed from a series of electron micrographs (n, nucleus). The black arrows indicate two pseudo-crystalline hemocyanin arrays that are magnified in the inserts with black frames. White frames indicate areas that are enlarged in (B-E). (**B**) Close-up of area B framed in (A), showing an elaborate extracellular lacuna (e) with slit apparatus (sa). m, enveloping lamina of extracellular matrix. Note the adjacent compartment with a pseudo-crystalline hemocyanin array (arrow) that seems to be accompanied by solitary hemocyanin didecamers. Coated vesicles (cv) are also present; they apparently lack hemocyanin. (**C**) Close-up of area C framed in (A), with rich rough ER (rer) and two pseudo-crystalline hemocyanin arrays in a compartment that seems to contain also free didecamers (arrow; see also inserts in (A)). (**D**) Close-up of area D framed in (C), featuring a small vesicle (v) within the extracellular lacuna (e). sa, slit apparatus; cv, coated vesicle. (**E**) Close-up of area E framed in (A), showing Golgi bodies (gb), mitochondria (mi) and many vesicles.

High pressure freezing in combination with the freeze substitution technique revealed greater detail of the rhogocytes (Fig 8A). Hemocyanin was mostly observed as pseudo-crystalline arrays surrounded by solitary didecamers (Fig 8B and 8C). In Fig 8D, a small vesicle within an extracellular lacuna is shown. A well-developed rough endoplasmatic reticulum, Golgi bodies and numerous mitochondria indicate extensive biosynthesis of protein, most likely hemocyanin (Fig 8E). Coated vesicles are also present (see Fig 8B and 8D); they appear to be either empty or filled with material which lacks the characteristic profiles of hemocyanin.

**Fig 9:**
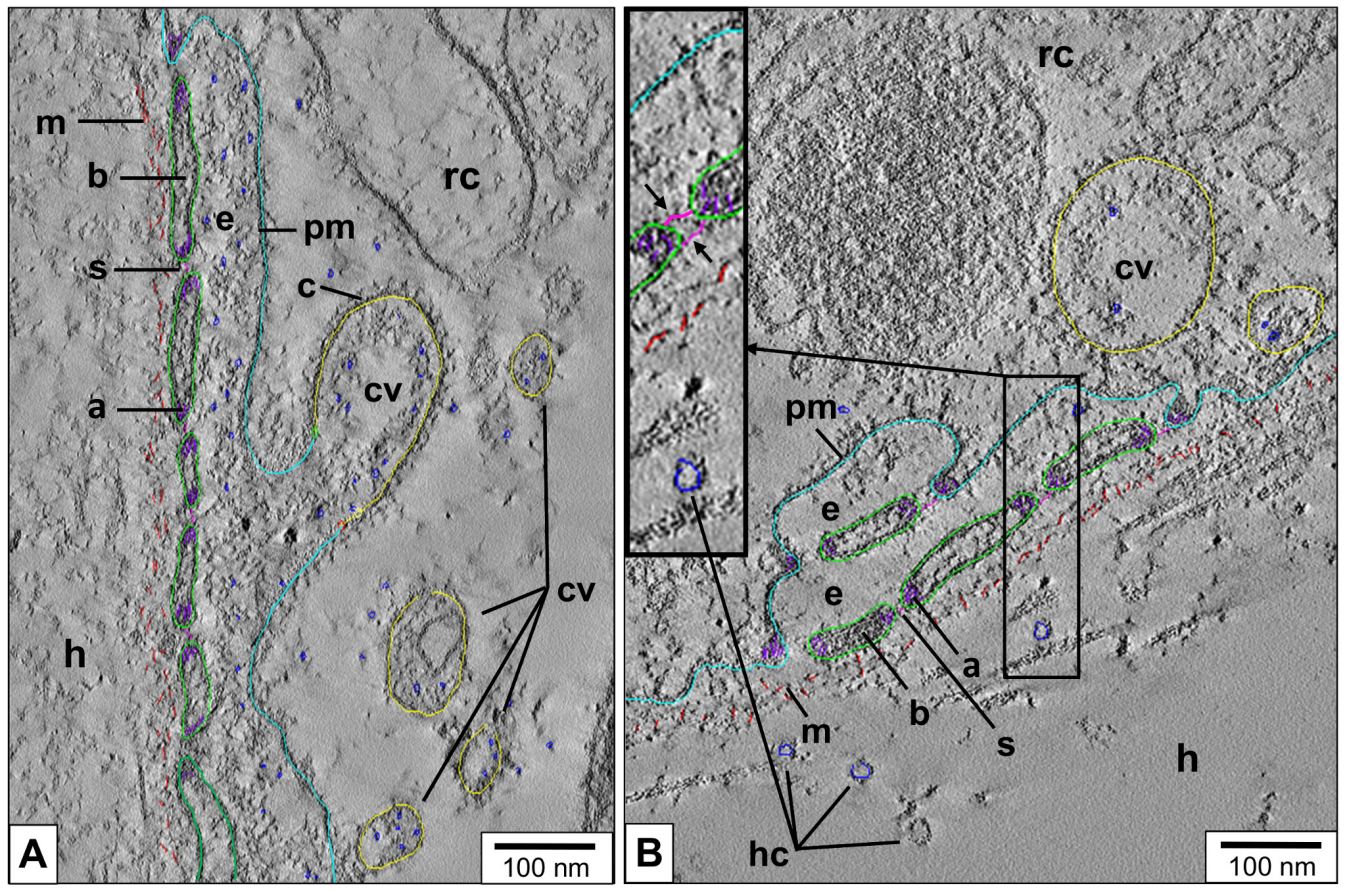
3D reconstruction of a rhogocyte region with slit apparatus. (**A,B**) Electron tomogram slices of two mantle rhogocytes (rc) and adjacent hemolymph spaces (h) of *L*. *stagnalis*, superimposed with their corresponding 3D reconstruction (calculated in IMOD). Note the enveloping lamina of extracellular matrix (m, red), plasma membrane (pm, cyan), extracellular lacuna (e), cytoplasmic bars (b, green), diaphragmatic slits (s, pink), coated vesicles (cv, yellow). Also note the highly dense, possibly actin-rich material in peripheral parts of the cytoplasmic bars (a, purple). (**A**) Note the large vesicle with clearly visible coat (c) and granular content (blue) in open contact with the extracellular lacuna. In the neighboring cytoplasm, confined coated vesicles are present. **(B)** A peculiar situation is seen here, with a seemingly doubled slit apparatus and two concatenated extracellular lacunae. The insert shows details of a diaphragmatic slit, with a double-layer slit diaphragm (black arrows); this resembles images from *B*. *glabrata* rhogocytes [3]. Several putative hemocyanin molecules (hc) are visible in the hemolymph space. Note that such structures are absent from the coated vesicles. Also note that they appear to be too large to pass through the present diaphragmatic slits.

3D reconstruction from tomogram slices of a region with slit apparatus shows, besides details of the cytoplasmatic bars and diaphragmatic slits, a coated vesicle fused with the plasma membrane (Fig 9A). In the vicinity, intracellular coated vesicles are present. The granular material within these vesicles, and within the adjacent extracellular lacuna, consists of particles that are much smaller than hemocyanin didecamers. In Fig 9B, several 25 nm rings outside of the enveloping lamina of extracellular matrix are shown that likely represent hemocyanin didecamers. Also in this case, the extracellular lacunae and the coated vesicles are devoid of hemocyanin. This suggests the following: (i) the rhogocytes shown in Fig 9 perform endocytosis, *via* coated vesicles, of the contents present in the extracellular lacunae; (ii) hemocyanin exocytosis does not occur in this cellular stage; (iii) exocytosis of hemocyanin *via* the slit apparatus as deduced from Fig 6 should then occur in a different cellular stage.

### Response of *L*. *stagnalis* rhogocytes to stress conditions

**Fig 10:**
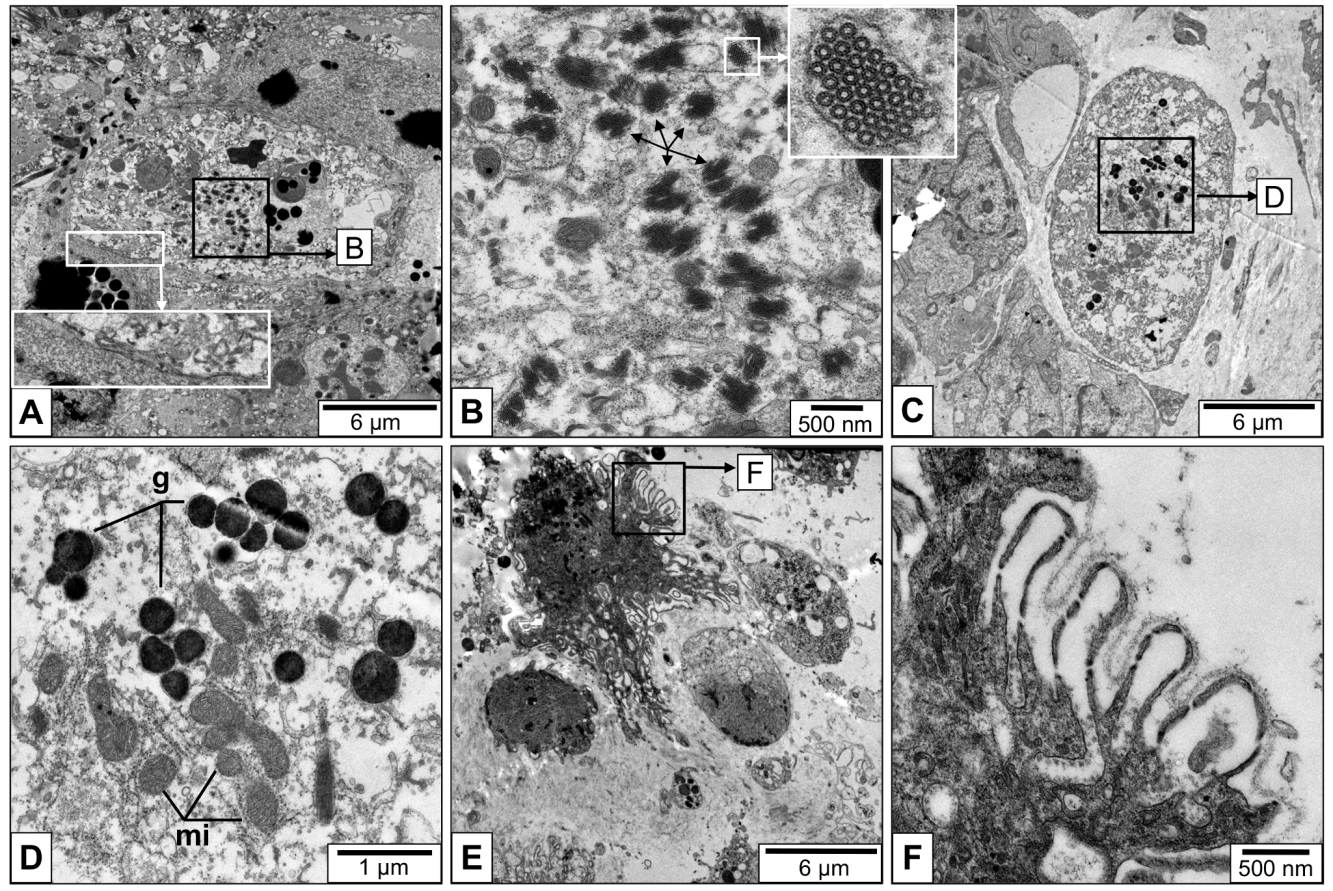
Response of rhogocytes to deprivation of food and cadmium stress. Electron micrographs of mantle tissue sections from *L*. *stagnalis* after starvation (A,B) and experimental cadmium contamination (C-F). (**A**) Total view of a rhogocyte from a snail deprived of food for 96 h. The insert (white box) shows that the cell surface is rather plain in that areas with invaginations and slit apparatus are rarely seen. The area within the black frame is magnified in (B). **(B)** Close-up of the indicated area in (A). Note the high number of small endomembrane compartments filled with a pseudo-crystalline hemocyanin array (arrows). The insert in (B) shows a further enlargement with top views of the hemocyanin stacks. (**C**) Total view of a rhogocyte from a cadmium-contaminated snail (48 h, 0.05mg/ml of CdCl_2_). The framed area is magnified in (D). (**D**) Close-up of the area framed in (C). Note many electron-dense granula (g) and mitochondria (mi). (**E**) A cluster rhogocytes from a cadmium-contaminated snail (96 h, 0.1mg/ml of CdCl_2_). The three small ovoid rhogocytes might stem from recent cell divisions. The larger rhogocyte with an irregular shape seems to be active in decontamination, as deduced from the framed area magnified in (F). (**F**) Close-up of the area framed in (E), showing a deeply folded cell surface with slit apparatus, suggesting exceptionally high ultrafiltration rates exhibited by this cell.

After deprivation of food for 96 h, individuals of *L*. *stagnalis* were fixed and the connective tissue was studied in the electron microscope. Compared to the controls, the rhogocytes of starved animals showed fewer areas with extracellular lacunae and cytoplasmic bars (Fig 10A). In the cytoplasm, the endoplasmic reticulum was still the most prominent structure. An unusually high number of small endomembrane compartments filled with pseudo-crystalline hemocyanin arrays was present (Fig 10B). We assumed that consequently, the hemolymph of starved animals should contain a higher hemocyanin concentration, and attempted to quantify this by measuring the protein content of hemolymph samples from 10 starved (1 week) and 10 control animals.

It turned out that this analysis was biased as follows: (i) The initial hemocyanin concentration in the hemolymph varied individually from 1.7 mg/ml to 7.4 mg/ml (average: *ca*. 3 mg/ml); (ii) seven days after the first bleeding, and probably stimulated by the latter, the hemocyanin concentration in the control animals had changed individually. In spite of these fluctuations, the average in both groups was still close to 3 mg/ml. It therefore appears that after seven days of starving, most of the additionally synthesized hemocyanin was still stored in the rhogocytes as pseudo-crystalline arrays.

In animals that survived for 96 h in water supplemented with 0.05–0.1mg/l cadmium chloride (*i*.*e*. experimental contamination), rhogocytes with high numbers of electron-dense granula and mitochondria were found (Fig 10C and 10D). The increase of electron-dense granula was quantified and found to be highly significant (Table 1). It appears that material (most probably cadmium chloride) is deposited during formation of the dense granula, and probably detoxified, and that this process is energy-consuming as deduced from the increased number of mitochondria. Moreover, clusters of rather small rhogocytes were frequently detected (Fig 10E), suggesting that upon cadmium contamination the number of rhogocytes is increased by cell division.

**Table 1:** Number of electron-dense granula in 15 individual rhogocytes from untreated control snails and CdCl_2_ exposed snails.

Controls (N = 15)			Cd^2+^ exposed (N = 15)		
4	2	2	8	15	16
3	4	4	11	8	28
4	3	0	7	16	8
4	2	8	7	11	9
0	1	1	9	19	28
Mean: 2.80; SD: 2.04; SEM: 0.53			Mean: 13.33; SD: 7.04; SEM: 1.82		

The two-tailed P value is < 0.0001, indicating high statistical significance.

SD, standard deviation; SEM, standard error of the mean.

Another major change was that compared to the controls, these rhogocytes exhibited many more extracellular lacunae, present in an otherwise not previously observed deeply folded manner (Fig 10E and 10F). In other words, the cell surface endowed with slit apparatus was significantly increased. This strongly suggests an uptake of the cadmium chloride through the molecular sieve constituted by the slit diaphragm.

## Discussion

### Rhogocyte structure and phylogeny

The present study confirmed previous electron microscopical investigations on *L*. *stagnalis* rhogocytes [2,5,15] with respect to their general tissue distribution and morphology. They are found either solitary or in small rhogocyte clusters in the connective tissue of foot and mantle, embedded between muscle cells, granular cells and secretory cells (see Figs 2 and 4). This resembles the situation in vetigastropods and other molluscs [1,4,8,12,16,17,27–31].

Rhogocytes were also detected in the heart and kidney of *L*. *stagnalis* [5], and in non-feeding developmental stages of a gastropod [32] Several authors proposed a connection between rhogocytes and the cyrtocytes of the protonephridial system [1,33,34] which was confirmed by a detailed electron microscopical study on gastropod larvae [24]. The typical slit apparatus of rhogocytes resembles that in insect nephrocytes and mammalian podocytes which both belong to the metanephridial system. Nephrocytes and podocytes show similar proteins such as nephrin and actin associated with the slit apparatus [6,7,35], and recently we collected evidence that both proteins are also present in the slit apparatus of *B*. *glabrata* rhogocytes [3]. The comparable results obtained here for *L*. *stagnalis* rhogocytes (see Fig 7) suggest that the principal morphology of rhogocytes, and notably of their slit apparatus, is conserved at least throughout the gastropods. The hypothesis of a common phylogenetic origin of podocytes, nephrocytes and rhogocytes is supported by the presence of actin and nephrin. Nevertheless, similar transcriptional gene profiles for development and function, and origin from similar progenitor cells would be much stronger arguments to conclude this case. Steward and coworkers [24] exemplified this for the connection of protonephridial terminal cells and rhogocytes.

### Biosynthesis of hemocyanin and hemoglobin

The conserved ultrastructure of rhogocytes does not necessarily mean that the biological functions of this cell type are strictly conserved. The expression of large amounts of a multi-domain hemoglobin by *B*. *glabrata* rhogocytes [3] is a prominent example of a novel function evolved by these cells. This hemoglobin is a characteristic feature of planorbid snails and apparently evolved from snail myoglobin [14]. Most other molluscs instead possess a multi-domain hemocyanin [for review, see [21]). By *in situ* hybridization, the present study confirmed the early hypothesis that in the pulmonate *L*. *stagnalis*, hemocyanin is synthesized by rhogocytes. Since this has been demonstrated already in case of the vetigastropods *Haliotis tuberculata* and *Megathura crenulata* [4,8], hemocyanin biosynthesis should be an ancient and basic rhogocyte function.

Both proteins, gastropod hemocyanin and planorbid hemoglobin, are based on exceptionally large, multi-domain polypeptide chains: 240 kDa with 13 consecutive heme domains in case of the hemoglobin, or 400 kDa with 8 concatenated functional units in case of the typical didecameric gastropod hemocyanin such as that from *L*. *stagnalis* (see Fig 1C) [14,36]. Members of the gastropod superfamily Cerithioidea even evolved a mega-hemocyanin with 550 kDa subunits encompassing 12 functional units [37,38]. Apart from the mammalian muscle protein titin, this is the largest polypeptide chain in the databases. Since the mutation likelihood of a protein increases with the length of its gene, most polypeptide chains are significantly smaller. Molluscan cells, notably the germline cells that transfer the genes across generations, seem to possess superior DNA repair mechanisms, and moreover a molecular machinery that supports gene duplication and gene fusion events. In this context it should be remembered that the genes encoding the two isoforms of *H*. *tuberculata* hemocyanin, although being separated for *ca*. 320 million years, have a completely conserved exon-intron structure [39], whereas the hemocyanin genes from other molluscs show a variety of late introns [40]. We are therefore convinced that rhogocytes possess some specific DNA control mechanisms still to be unraveled.

In electron micrographs of *L*. *stagnalis* tissue sections, individual hemocyanin molecules are clearly identified in the hemolymph and in rhogocytes by their typical top and side views, and are present in abundance. In ER cisternae, they often form pseudo-crystalline arrays composed of stacked didecamers (see Fig 5). This has also been observed, under special experimental conditions, with purified hemocyanin [23] and allows a clear identification as hemocyanin because due to this molecular superposition, in top views of such stacks more structural details emerge (see Fig 5E). However, there is a discrepancy concerning the diameter of the hemocyanin particles when viewed from the top: In solution it is *ca*. 35 nm as deduced from 3D cryo-electron microscopy [19,20] and negatively stained purified hemocyanin (see Fig 5A, and literature cited in [21]). In contrast, in electron micrographs of tissue sections, we and other authors measured only 25 nm diameter for hemocyanin didecamers (see Fig 5 and [17,24]). A considerable shrinking of the molecules under the applied fixation and embedding conditions is therefore suggested, and might be explained by the loss of water.

*B*. *glabrata* hemolymph contains traces of a truncated hemocyanin [14,21]. According to electron micrographs of the negatively stained protein, it is a single decamer 35 nm in diameter, lacking the internal collar structure characteristic for didecameric gastropod hemocyanins (for review, see [21]). This lack of a collar allows a considerable flattening of side views of the *B*. *glabrata* hemocyanin particles in negatively stained electron microscopical samples [14,21]. Candidate stacks of hemocyanin particles have been detected in ER cisternae of *B*. *glabrata* rhogocytes [3], and except for the lack of an internal collar, the top views of such stacks closely resemble the hemocyanin stacks in *L*. *stagnalis*. Thus, there is little doubt that these structures in *B*. *glabrata* are indeed hemocyanin, but their particle diameter is 50 nm instead of 25 nm. Why this truncated hemocyanin appears to swell under the same fixation conditions that cause typical hemocyanin didecamers to shrink remains unclear.

Furthermore, it is quite remarkable that according to the observed increase in the number of vesicles containing pseudo-crystalline hemocyanin arrays, deprivation of food for several days yielded an up-regulation of hemocyanin synthesis. This was first observed in *B*. *glabrata* [3] but was much more obvious in *L*. *stagnalis*, due to the much larger amount of such hemocyanin arrays [see Fig 10B]. The metabolic meaning of this up-regulation of hemocyanin, and the source of the necessary amino acids remain obscure. During starvation of *Drosophila* larvae an up-regulation of hemolymph proteins was observed [41]. Insect larvae in general use derivatives of hemocyanin, termed hexamerins, as amino acid storage proteins during metamorphosis [42]. This, however, concerns a different animal phylum and a convergently evolved hemocyanin superfamily. In molluscs, studies on the hemocyanin metabolism are scarce [43]. Another point that puzzles us is the question whether in *B*. *glabrata*, the hemoglobin as the major hemolymph protein is also upregulated during starvation, or whether this effect is restricted to hemocyanin. If the latter is the case, how should the tiny overall amounts of the truncated hemocyanin in *B*. *glabrata* fullfill the same metabolic role in starving animals as the abundant didecameric hemocyanin in *L*. *stagnalis*?

### Heavy metal uptake

Cadmium is a very toxic heavy metal often used in industry, notably in the context of electroplating. It can be a serious pollutant in freshwater ecosystems where it accumulates through the food chain. Due to its low permissible exposure limit, even trace quantities can lead to overexposures. Effects of heavy metal exposure of *L*. *stagnalis* and *B*. *glabrata* on growth, mortality and reproduction have been documented [44–49]. A role of rhogocytes in detoxification of heavy metals has been proposed (*e*.*g*. [12,13,50]).

**Fig 11:**
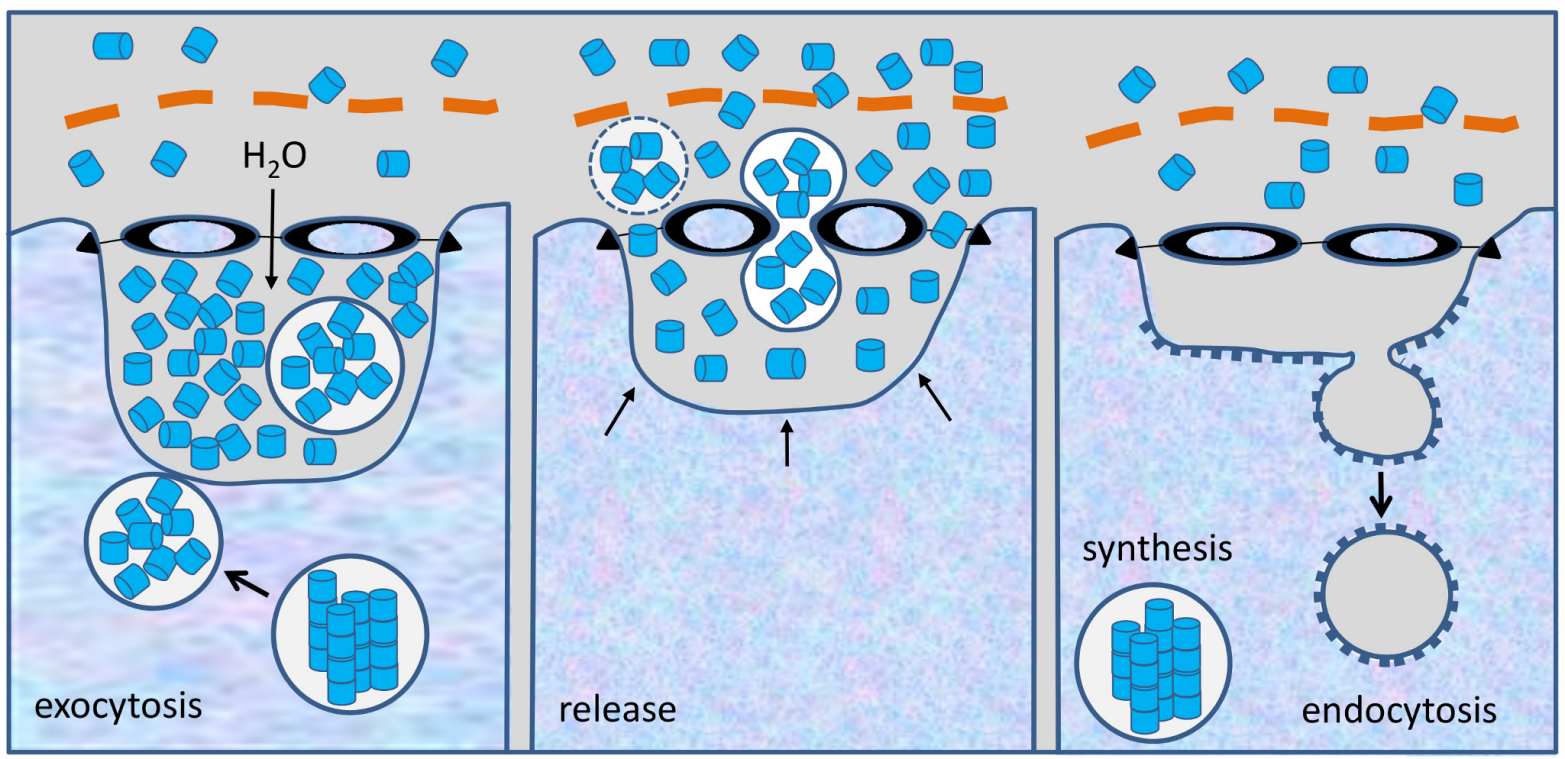
Hypothesis for the passage of material through the slit apparatus. We assume that there are three different cellular stages: (**1**) Uncoated vesicles transport newly synthesized hemocyanin stacks to the plasma membrane; the stacks dissociate into single didecamers; exocytosis and accumulation of hemocyanin within the extracellular lacunae (their further release into the hemolymph is blocked by the slit apparatus); colloidosmotic pressure in the lacunae increases; water loss of the cytoplasm through exocytosis and enlargement of the extracellular lacunae yields the wrinkled rhogocyte population (observed by Sairi and coworkers [52]). **(2)** Pressure-induced contraction of the actin-rich cytoplasmic bars yields enlargement of the diaphragmatic slits. Free hemocyanin and occasional vesicles are released through the slits and the enveloping lamina of extracellular matrix (orange) into the hemolymph. The extracellular lacunae shrink. (**3**) Endocytosis of solvent by coated vesicles, with uptake of heavy metal ions; passage of hemocyanin through the slits is blocked again by the relaxed bars which prohibits its uptake from the hemolymph; the cell regains its original water content; the extracellular lacunae shrink further (ovoid rhogocyte population); hemocyanin biosynthesis starts again.

Indeed, *B*. *glabrata* rhogocytes show specific reactions if the animals are exposed to cadmium-contaminated tank water: (i) Increased number of rather small rhogocytes, apparently the result of increased cell division; (ii) increase of the cell surface by intensive folding which primarily enlarges the total surface of the slit apparatus; (iii) increase in the number of electron-dense granula and probably mitochondria [3]. A comparable reaction was observed in *L*. *stagnalis* (see Fig 10). This underlines the existence of a heavy metal detoxification pathway leading from diffusion or pressure filtration of cadmium-containing hemolymph through the slit apparatus into the extracellular lacunae, endocytosis by coated vesicles, and storage and possible detoxification of the heavy metal in electron-dense granula (Fig 11).

We assume that this putative ultrafiltration by the slit diaphragm excludes hemocyanin (or hemoglobin) molecules to pass from the hemolymph through the slit apparatus. This concept is supported by the observation that in coated vesicles, hemocyanin particles seem to be absent (see Fig 9). It is also an argument against the possibility that a major role of rhogocytes is sequestering hemocyanin molecules taken up from the hemolymph, a hypothesis proposed by several authors [5,16–18].

A correlation exists between the level of animal exposure and the degree of structural changes of rhogocytes: the higher the applied concentration of cadmium, the more intense the changes in cell structure. Even in an almost unpolluted environment, snails concentrate cadmium in their midgut gland, and detoxify it by using a specific metallothionein isoform [51]. Upon heavy metal stress, these snails show significant changes in the ultrastructure of their rhogocytes. Therefore, they might be considered as bioindicators of metal pollution in freshwater systems.

### Exocytosis of hemocyanin, and role of the slit apparatus: a model

Very recently, by flow cytometry and cell sorting, Sairi et al. [52] isolated two different rhogocyte populations from mantle tissue of the vetigastropod *Haliotis laevigata*: ovoid *versus* irregularly shaped cells. By simultaneous *in situ* hybridization and anti-hemocyanin immunolabeling, they discovered in the confocal microscope that both cell populations synthesize the same hemocyanin isoform, and accumulate it in the cell periphery. The irregularly shaped cells exhibited strong antibody labeling signals and punctuate mRNA probe signals around the nucleus. The ovoid cells showed lower antibody signals and widely distributed mRNA signals. In the cell periphery, the antibody labelling was often visible as separate fluorescent dots [52], which might represent large vesicles such as those in Fig 6A–6D, and/or extracellular lacunae filled with hemocyanin. (These fluorescent dots separated by black background are addressed by these authors as “pore structures”. This, however, is a misleading term for these compartments, because the actual diaphragmatic pores are much smaller.)

From their data, Sairi and coworkers [52] concluded that rather than functioning as a molecular sieve, the slit apparatus acts as a barrier that traps the newly synthesized hemocyanin molecules for a while within the extracellular lacunae. (And we would add that simultaneously, the slit apparatus prevents that “old” hemocyanin from the hemolymph entering these lacunae.) According to this hypothesis, vesicles transport the freshly synthesized hemocyanin to the cell periphery, and then release their watery content into the extracellular lacunae. This causes the extracellular lacunae to expand and the cytoplasm to shrink, resulting in a wrinkled-like overall cell surface. Thereafter, “something” stimulates the cells to stretch back to their original size and ovoid shape which presses the fluid, together with the hemocyanin, from the extracellular lacunae through the thereby widened diaphragmatic slits into the hemolymph. Subsequently, in the ovoid cell population, new mRNA is transcribed and released into the cytoplasm, and the circle starts again [52].

In our previous paper on *B*. *glabrata* rhogocytes, we frequently observed protein-containing uncoated vesicles close to the plasma membrane. Sometimes, we found such vesicles within an extracellular lacuna, between lacuna and enveloping lamina of extracellular matrix, and even in the process of passing through a diaphragmatic slit [3]. This stimulated us to propose that in *B*. *glabrata*, the release of the freshly synthesized hemoglobin into the hemolymph occurs by vesicle passage through the slits (see Fig 11), caused by a “signal-induced” widening of the slits under contribution of the adjacent actin bundles [3]. However, a weakness of this hypothesis is that in the present study on *L*. *stagnalis*, we often observed free hemocyanin particles in the extracellular lacunae, whereas extracellular vesicles are very rarely seen. This supports the release model proposed by Sairi and coworkers [52], which has the additional advantage that this kind of release might function not only with free hemocyanin molecules, but also with small occasional vesicles (see Fig 8D).

A weakness of the release model of Sairi and coworkers [52] is that it does not explain the mechanism of the release stimulus, and more importantly, it neglects how after exocytotic activity the rhogocyte regains its original water content which then should push the contents of the extracellular lacunae through the widened diaphragmatic slits.

We incorporated the release concept of Sairi and coworkers [52] in a modified form into our own release model (see Fig 11). Our idea is that the colloidosmotic pressure inflicted by the heavily accumulating hemocyanin molecules causes a water flow from the hemolymph into the extracellular lacunae. They swell to a certain pressure level that finally opens the diaphragmatic slits like floodgates. This opening might be caused by contraction of the actin-rich peripheries of the cytoplasmatic bars, keeping in mind that single unit-type smooth muscles, as for example those in the gastropod foot, show automatic myogenic activity that is increased by stretch. As discussed previously, the proteins constitution the slit diaphragm should allow a temporary enlargement of the sieve pores (see Discussion in [3]). After release of the hemocyanin-containing solvent into the hemolymph, the “floodgates” regain their original size. Subsequently, the rhogocyte gathers water by resuming endocytotic activity, thereby regaining its ovoid shape. Hemocyanin-specific mRNA is newly transcribed, and the hemocyanin biosynthesis starts again.

Thus, the important results of Sairi and coworkers [52] in combination with our previous [3] and the present electron microscopical data provide the first convincing model of how freshly synthesized hemocyanin (respectively hemoglobin in planorbids) is released through the slit apparatus into the hemolymph (see Fig 11). It also explains why rhogocytes require a slit apparatus, whereas other secretory cells release their products directly into the environment: Besides hemolymph protein production, rhogocytes have additional important functions, notably the uptake of heavy metals from the hemolymph. The extracellular lacunae provide a compartment from which the cell is able to collect, by endocytosis, water and small particles such as heavy metal ions, without simultaneously gathering hemocyanin from the hemolymph. Hemocyanin and other large hemolymph proteins are excluded from the extracellular lacunae by the sieving effect of the slit apparatus. This model requires that during endocytotic activity, hemocyanin exocytosis is discontinued, and *vice versa*. The two different rhogocyte populations observed by Sairi and coworkers [52], the absence of hemocyanin in extracellular lacunae with endocytotic activity (see Fig 9), and the abundance of hemocyanin in these lacunae in other phases (see Fig 6) support this model.

## Materials and Methods

### Ethics statement

The snail *L*. *stagnalis* is very abundant in Germany and Central Europe and neither an endangered nor a protected species. It was collected in a local pond on private land belonging to one of the authors (JM). Therefore, no specific permissions were required for these locations and activities. The animals were maintained in controlled freshwater tanks at 20°C. All animal work has been conducted according to national guidelines. Animals were killed under 7% magnesium chloride/ice water anesthesia. There was no need for involving an ethics committee in the case of these gastropods.

### Paraffin sections and light microscopy

Sacrificed individuals of *L*. *stagnalis* were removed from their shell, fixed in formalin, dehydrated in a series of increasing alcohol concentrations followed by xylol, and finally embedded in paraffin. Using a rotation microtome (Leica Microsystems GmbH, Wetzlar, Germany), 3–5 μm sections were prepared, stained with hematoxylin/eosin and observed in the Nikon Eclipse 80i histological microscope (Nikon, Tokyo, Japan), equipped with a Nikon DS-Fi1 digital camera and a digital sight control unit (Nikon, Tokyo, Japan). Two subsequent sections were stained with Movat´s pentachrome and azan [53–55], respectively. The next two sections remained unstained for further use in *in situ* hybridization and immunohistochemistry. Total scans (one single image composed of 100 individual images) of 2500x1200 pixels were also generated by using an automatic scanning table (Prior scientific, Rockland, USA).

### Immunohistochemistry

The MaxLSABTM rabbit HRP detection kit (Max Vision Biosciences Inc., Washington, USA) was used according to the protocol provided by the company. Rabbit primary antibodies against *Aplysia californica* hemocyanin [40] were diluted 1:35,000 with 50mM Tris/HCl pH 7.6. The color reaction was stopped after 10 minutes. In pre-experiments, the antibody concentration was optimized to avoid non-specific binding.

### Tissue preparation and electron microscopy

Pieces of *ca*. 3 mm^3^ were extracted from mantle and foot and fixed for electron microscopy as described [3]. Ultrathin sections (80–100 nm) were prepared on an ultramicrotome (Reichert Ultracut E, Leica Microsystems) and collected on grids covered with formvar film. They were stained with 2% uranyl acetate in 50% ethanol for 10 minutes and lead citrate for 2 minutes [56]. The sections that would be further used for tomography were incubated with 10nm gold particles for 5 minutes on each side. Images were collected on a CCD (charge-coupled device) camera (TVIPS, Gauting) operated on a Tecnai12 transmission electron microscope at 120kV. The cells were viewed in 3D as described [57,58]. The tilt series were recorded at a magnification of 23,000x binned by 2 (2048x2048 pixels micrographs), over a tilt range of -60° to +60° with 1.5° increment, using the EMMENU4 software (TVIPS, Gauting).

### Alignment and 3D reconstruction

Each set of TIFF image files was converted into a single.mrc file using IMAGIC-4D software [59]. Alignments and 3D reconstructions of raw tilt series using 15–30 fiducials were done with IMOD under eTomo [60]. A semi-automated procedure using the module “e2boxer” implemented in the EMAN2 software package was used to count the electron-dense granula in electron micrographs [61].

### Stress conditions

The animals were kept in tap water for 96 h in the dark, deprived of food. Other individuals were fed normally, but they were exposed for 12, 48 and 96 h to nominally assigned cadmium contaminations (0.05mg/l or 0.1mg/l of CdCl_2_). The fixation of tissues for electron microscopy was done as described above.

### Purification and electron microscopy of *L*. *stagnalis* hemocyanin

Hemolymph collection, hemocyanin purification and negative staining electron microscopy of hemocyanin were done as described for other molluscan hemocyanins [62]. As negative stain, 1% uranyl acetate was used [63].

### Tissue preparation and immunogold labeling

Small pieces from snail mantle tissue were extracted and fixed for immunoelectron microscopy as described [3]. The material was embedded in LR-White and polymerized in UV-light as previously described [64]. The monoclonal mouse anti-actin antibody 05 (Thermo Scientific, Schwerte, Germany) and polyclonal rabbit anti-*Aplysia* hemocyanin antibodies [40] were used. Nanogold-coupled anti-mouse secondary antibodies were applied. Nanogold-labeling was silver-enhanced [65]. The sections were contrasted with 2% uranyl acetate and lead citrate [56], and further analyzed by electron microscopy.

### Immunofluorescence microscopy on frozen tissue sections

The procedure was done according to Schaffeld & Markl [66]. Frozen tissue sections of 7 μm thickness were prepared on a cryotome HM 500 OM (Microm, Walldorf, Germany). They were incubated with guinea pig anti-nephrin antibodies NPHN/NPHSI (Acris Antibodies GmbH, Herford, Germany) diluted 1:50 in PBS, and with mouse monoclonal anti-actin antibody C4 (Seven Hills Bioreagents, Cincinnati, USA) diluted 1:150 in PBS. The sections were washed and incubated with the (diluted 1:200 in PBS) Texas Red-coupled goat-anti-guinea-pig and Cy2-coupled goat-anti-mouse (Dianova, Hamburg, Germany) secondary antibodies, respectively. The sections were studied at the Leitz DM RBD fluorescence microscope (Leica, Wetzlar, Germany).

### *In situ* hybridization

30 mg of *L*. *stagnalis* tissue was distrupted and the RNA was extracted according to the Total RNA isolation NucleoSpin RNA II Kit (Macherey-Nagel). Specific primers were designed (forward primer: CCGAGGCTATCCGCAAGGGC and reverse primer: GGTGTGCACGACACCGAGGG) and generated by Sigma-Aldrich (Hamburg, Germany), based on the DNA sequence of the C-terminal and early tail region of functional unit FU-h of the *L*. *stagnalis* hemocyanin (source: unpublished transcriptome data kindly provided by Prof. Hisayo Sadamoto, Tokushima Bunri University, Kagawa, Japan). Specific cDNA probes were generated by PCR (Super script III first-strand synthesis system for RT-PCR, Invitrogen, Darmstadt). The size of the product was tested by gel electrophoresis. The DNA fragment was extracted from the gel according to the GeneJET ^TM^ gel extraction kit (Fermentas, St. Leon-Roth), inserted into a vector and transformed in bacterial cells (StrataClone ^TM^ PCR Cloning Kit, Agilent, Böblingen).

The cells were applied to agar plates with antibody resistance and incubated at 37°C overnight. The white colonies were selected, allowed to grow in Lysogeny broth (LB) medium. Afterwards, the GeneJet^TM^ Plasmid Miniprep Kit (Fermentas, St. Leon-Roth) was used to isolate the plasmid DNA. The size of the products was checked with PCR and gel electrophoresis. The DNA probes were digoxigenin-labeled (DIG-labeling Kit, Roche, Mannheim, Germany).The *in situ* hybridization on paraffin sections and whole mounts were carried out according to published protocols [4,67], both with the modifications described recently [3].

### High pressure freezing and freeze substitution

Tissue samples were frozen under high pressure, namely 2100 bar [68]. The tissue (fresh and up to 200 μm thick) was enclosed and protected in a small volume between two specimen carriers (aluminium type A specimen carrier, 100 μm/200 μm cavity; aluminium type B specimen carrier, flat/300 μm cavity) and locked inside the specimen pressure chamber. Dextran (20% in water) was used as a cryo-protectant. Both carriers were dipped in hexadecene. Liquid nitrogen was used as cooling medium. To ensure high quality preservation of biological samples, the high pressure freezing technique was combined with freeze substitution and resin embedding. This includes dehydration of the cryo-fixed sample at -90°C by substituting the ice for an organic solvent. More detailed, the cryo-fixed samples were put at -90°C into the substitution medium (0.1% uranyl acetate + 0.2% osmium tetroxide + 5% water in acetone), (EM, AFS, Leica Microsystems, Germany) for 2 days. The samples were further rinsed in pure acetone (3 x 5 min) and infiltrated in EPON (acetone:EPON 3:1 for 2 h, 1:1 for 2 h, 1:3 for 2 h and pure EPON overnight). The next day, the samples were infiltrated with new pure EPON and further polymerized at 60°C for 48 h. Ultrathin sections were prepared as described above.

## Abbreviations

3D: three dimensional
ER: endoplasmic reticulum
